# *CYP2D6* ‐Guided Opioid Therapy for Chronic Pain: A Multi‐Center Pragmatic Randomized Controlled Trial

**DOI:** 10.1002/cpt.70505

**Published:** 2026-10-15

**Authors:** Todd C. Skaar, Rachel A. Myers, Roger B. Fillingim, John T. Callaghan, Kerri L. Cavanaugh, Hrishikesh Chakraborty, Jordan F. Baye, Emily J. Cicali, Michael T. Eadon, Erica N. Elwood, Elizabeth C. Harris, Lindsay J. Hines, Khoa A. Nguyen, Michelle A. Ramos, Marc Rosenman, Azita Sadeghpour, Saskia Shuman, Rajbir Singh, Emma M. Tillman, Christina M. Wyatt, Carol R. Horowitz, Deepak Voora, Lori A. Orlando, Josh F. Peterson, Larisa H. Cavallari, Julie A. Johnson, Paul R. Dexter, the IGNITE Pragmatic Trials Network, L Orlando, D Voora, C Horowitz, C Horowitz, N Calman, T Skaar, T Skaar, P Dexter, T Skaar, P Dexter, J Johnson, L Cavallari, J Johnson, L Cavallari, J Johnson, L Cavallari, K Blake, K Blake, K Blake, J Peterson, J Peterson, K Cavanaugh, R Singh, R Singh, L Hines, C Wyatt, H Chakraborty, Christina Wyatt, Geoff Ginsburg, Kady‐Ann Steen‐Burrell, Bhargav Adagarla, Sarah George, Beth Harris, Jaclyn Holland, Yashika Johnson, Phyllis Kennel, Kristen Linney, Rania Metry, Rachel Myers, Wanda Parker, Gayle Passmore, Carol Pereira, Teji Rakhra‐Burris, Ryanne Wu, Ruth Lehan, Tyffany Locklear, Carol Horowitz, Michelle Ramos, Sabrina Clermont, Bart Ferket, Kenneth Fifer, Diane Hauser, Joseph Kannry, James Murrough, Aniwaa Owusu Obeng, Janet Seo, Tatiana Sabin, Nandini Shroff, Paul Dexter, Abi Colwell, Amy M. Breman, Zeruesenay Desta, Cathy Fulton, Jennelle C. Hodge, Sheryl Lynch, Jonathan Oliver, Victoria M. Pratt, Ross Robinson, Elizabeth Rowe, Jennifer Stuart, Emma Tillman, Ashley Vetor, Julie Johnson, Larisa Cavallari, Alexander Parker, Erica Elwood, Kathryn Blake, Emily Cicali, Kelsey Cook, Karam Diaby, Julio Duarte, Ben Duong, Karla Giron, Elizabeth Eddy, Julia Krutov, Carol Mathews, Haesuk Park, Robyn Nelson, Ryan Rhoden, Caroline Schlierle, Taylor Sullivan, Almut Winterstein, Kristen Wiisanen, Josh Peterson, Kerri Cavanaugh, Sara Van Driest, Sara Block, Jordan Baye, Michelle Benck, Chantel Bender, Sylvia Eluhu, Colette Free, Shane Gonnelly, Sarah Hedeen, Lindsay Hines, Steve Houtschilt, Michelle Liu, Salisha Marryshow, Nicole Neville, Natasha Petry, Sidd Pratap, Vernon Sherden, Kimberly Snell, Ana Tomescu, Megan Trietsch, James Walker, Deanna Web, Erin Whiting, Russell A. Wilke, Simona Volpi, Sarah Hutchison, Natalie Kucher, Ebony Madden, Jessica Reinach, Renee Rider, Ismail Safi, Ella Samer, Lori Orlando, Lani Banez, Lisa Bendz, Jackie Jordan, Ann McGee, Christina Nix, Lorraine Vergara, Tyler Colon, Aaishah Francis, Samantha Guagliardo, Najiba Khan, Camila Tan Lam, Michelle Sciarrino, Emma Maiman‐Stadtmauer, Sofia Medina‐Pardo, Ololade (Lola) Williams, Sheng Luo, Kevin Anstrom, Alicia Ellis, Stephen Ellis, Hwasoon Kim, Peter Merrill, Lilin She, Jennifer Shepherd, Saira Siddiqui, Jun Wen, Brittany Davis, John Howard, Alia Jamison, Lydia Joyner, Ellie Kiihne, Johnathan Oliver, Libbie Silverman, Jordan Sonneville Jennifer Stuart, Laurel Tokar, Neil Calman, Daviana Buck, Nicole Canchucaja Porshia Cook, Ariel Jacobs Danielle John, Mirta (Mimi) Milanes, Wambui Ngari, Megan Triestch, Abraham Garcia, Carol Gutierrez, Steven Houtschilt, Maria Lopez, Alan Mejia, Anaite Montes Bu, Deanna Webb, Sonja White, Ali Brewer, Brittni Deadrick, Alexis Meyer, Blair Svennes, Hana Al Alshaykh, Tala Basha, Asia Cobb, Brendon Cooper, Amanda Elchynski, Amanda Elsey, Anuksha Gotmare, Lauren Lemke, Arielle Nelson, Michael Read, Genevieve Rosier, Isabella Schultz, Chiara Chermont Spina, Joshua Terrell, Alexa Valko, Precious Williams, Tracy Leonard, David Smith, Marian Davis, Vincent Guerra, Jennifer Salo, Claudia Cartaya Torres, Jorge Valedon, Paola Nieves Viana, Jose Alonso, Jeffery Gainer, Morgan Howard, Fatoumata Kaba, Brianna Rivers, Liliana Serrano, Kimberly Vigal, ADeia Williams, Angelo Lazaro, Salisha Merryshow, Henry Ong, John Thomas Callaghan, Raheleh Rahimi Darabad, Daniel Getu, Katherine Huber, Judive Jocelyn, Robin Morman Li, Rosalena Muckle, Brandi Plunkett, Siegfried Schmidt, Evgenia Teal, Petra Townsend, Joshua R. Wellington

**Affiliations:** ^1^ Division of Clinical Pharmacology, Department of Medicine Indiana University School of Medicine Indianapolis Indiana USA; ^2^ Department of Medicine, Clinical Research Unit Duke University School of Medicine, Duke University Durham North Carolina USA; ^3^ Department of Community Dentistry and Behavioral Science University of Florida College of Dentistry Gainesville Florida USA; ^4^ Division of Nephrology & Hypertension Vanderbilt University Medical Center Nashville Tennessee USA; ^5^ Veterans Health Administration VA Tennessee Valley Health Care System Geriatric Research, Education and Clinical Center (GRECC), and VETWISE‐LHS Center of Innovation Nashville Tennessee USA; ^6^ Department of Biostatistics and Bioinformatics Duke University Durham North Carolina USA; ^7^ Department of Pharmacy Practice South Dakota State University Brookings South Dakota USA; ^8^ Sanford Imagenetics, Sanford Health Sioux Falls South Dakota USA; ^9^ Department of Pharmacotherapy and Translational Research and Center for Pharmacogenomics and Precision Medicine, College of Pharmacy University of Florida Gainesville Florida USA; ^10^ Division of Nephrology Indiana University School of Medicine Indianapolis Indiana USA; ^11^ Duke Clinical Research Institute Duke University School of Medicine Durham North Carolina USA; ^12^ Department of Psychology University of North Dakota Grand Forks North Dakota USA; ^13^ Institute for Health Equity Research Icahn School of Medicine at Mount Sinai New York New York USA; ^14^ Department of Pediatrics Indiana University School of Medicine Indianapolis Indiana USA; ^15^ Ann & Robert H. Lurie Children's Hospital of Chicago and Feinberg School of Medicine, Northwestern University Chicago Illinois USA; ^16^ Duke Precision Medicine Program, Department of Medicine Duke University Durham North Carolina USA; ^17^ The Institute for Family Health New York New York USA; ^18^ Department of Internal Medicine Meharry Medical College Nashville Tennessee USA; ^19^ Division of Nephrology, Department of Medicine Duke University School of Medicine Durham North Carolina USA; ^20^ Department of Biomedical Informatics Vanderbilt University Medical Center Nashville Tennessee USA; ^21^ Clinical and Translational Science Institute The Ohio State University Columbus Ohio USA; ^22^ Department of Internal Medicine, College of Medicine The Ohio State University Columbus Ohio USA; ^23^ Department of Pharmaceutics & Pharmacology, College of Pharmacy The Ohio State University Columbus Ohio USA; ^24^ Regenstrief Institute, Inc. Indianapolis Indiana USA; ^25^ Sidney & Lois Eskenazi Hospital and Eskenazi Health, Inc. Indianapolis Indiana USA; ^26^ Department of Medicine Indiana University School of Medicine Indianapolis Indiana USA; ^27^ Present address: Wake Forest University School of Medicine Winston‐Salem North Carolina USA

## Abstract

The analgesic effectiveness of several opioids depends on CYP2D6‐mediated metabolism to more active opioid receptor agonists. CYP2D6 activity is impacted by genetic variability and medications that inhibit CYP2D6. We conducted a pragmatic, multi‐center, randomized, controlled trial to determine whether CYP2D6‐guided opioid prescribing improves chronic pain. Participants were adults recruited from primary care or pain specialty clinics with at least 3 months of chronic pain treated or considered for treatment with tramadol, hydrocodone, or codeine. Participants were randomized to the usual care or CYP2D6‐guided arm, in which *CYP2D6* pharmacogenetic testing was performed. Healthcare professionals were recommended to avoid tramadol, hydrocodone, or codeine in patients with CYP2D6 intermediate or poor or ultra‐rapid (for safety) metabolizer phenotypes based on either genotype or drug interactions. The primary outcome was the change in composite pain intensity scores from baseline to 3 months. Of 1048 enrolled participants, 282 had an actionable CYP2D6 phenotype. In this group, CYP2D6‐guided recommendations did not improve the change in composite pain intensity scores from baseline to 3 months compared with usual care (mean [SD] −0.8 [2.4] vs. −0.7 [2.3]) or improve drug‐gene concordance (26.8% vs. 27.1%) in the CYP2D6‐guided versus usual care arm, respectively. In an exploratory analysis, participants whose therapy changed to align with their CYP2D6 phenotype had significantly improved pain scores. In conclusion, incorporating CYP2D6‐guided recommendations for opioid therapy into routine practice did not change prescribing sufficiently to assess its impact on chronic pain control, suggesting that EHR‐integrated CYP2D6 guidance is insufficient to change opioid prescribing for chronic pain.

**Study Highlights**

**WHAT IS THE CURRENT KNOWLEDGE ON THE TOPIC?**

Several opioids are metabolized to more active metabolites by CYP2D6, and impaired metabolism via CYP2D6 has been shown to reduce opioid efficacy. It is unclear whether providing the patients' *CYP2D6* genotype and phenotype information impacts pain control in the real‐world setting.

**WHAT QUESTION DID THIS STUDY ADDRESS?**

Does providing *CYP2D6* genotype‐ and phenotype‐guided recommendations (based on both genotype and CYP2D6‐interacting drugs) to healthcare professionals improve pain control in patients with chronic pain?

**WHAT DOES THIS STUDY ADD TO OUR KNOWLEDGE?**

Our findings suggest that providing electronic health record (EHR)‐integrated CYP2D6 guidance without expanded healthcare professional support may be insufficient to change opioid prescribing. An exploratory analysis found significantly improved pain scores among participants who had drug therapy changed to align with their CYP2D6 phenotype. Our work suggests that there are many challenges to convincing prescribers and/or patients to change existing opioid therapy, and thus pharmacogenetic implementation may be most successful when utilized at the time of opioid therapy initiation.

**HOW MIGHT THIS CHANGE CLINICAL PHARMACOLOGY OR TRANSLATIONAL SCIENCE?**

Our results indicate that more work needs to focus on the implementation strategies for using genotype‐informed CYP2D6 metabolizer status to drive opioid selection in real‐world patients with chronic pain.

Chronic pain reduces the quality of life for millions of individuals in the United States (US) and costs over $700 billion annually.^1^ Approximately 20% of adults in the US experience pain most days or every day.^2^ Opioids are frequently used to treat chronic pain despite risks of dependence, overdose, and diversion.^3^ Moreover, the clinical response to opioids among patients with chronic pain shows substantial interindividual variability.^4^, ^5^, ^6^, ^7^, ^8^ This variability appears to be due, in part, to the variable metabolism of tramadol, codeine, hydrocodone, and oxycodone by hepatic cytochrome P450 2D6 (CYP2D6) to active metabolites, some of which are up to 100‐fold more active agonists of the opioid receptor than the parent compounds.^5^, ^9^ The *CYP2D6* gene has > 180 known alleles that result in individuals having normal metabolizer (NM), ultrarapid metabolizer (UM), intermediate metabolizer (IM), or poor metabolizer (PM) phenotypes,^10^ with the IM and PM phenotypes resulting in the generation of little or no active opioid metabolites and the UM phenotype generating increased and potentially unsafe metabolite levels. Several studies have shown reduced analgesia in individuals carrying decreased or no‐function *CYP2D6* alleles.^5^, ^9^ In addition to genetic variants, concurrent use of strong CYP2D6 inhibitors eliminates most CYP2D6‐mediated metabolism, and moderate inhibitors substantially reduce CYP2D6 metabolism with a proportionate impact on active opioid metabolites.^11^, ^12^ Strong and moderate CYP2D6 inhibitors include several commonly used antidepressants. Because chronic pain frequently co‐occurs with depression,^13^ many patients with chronic pain are prescribed antidepressants that inhibit CYP2D6, reducing opioid bioactivation and presumably effectiveness.^14^, ^15^, ^16^, ^17^

Published guidelines facilitate the use of *CYP2D6* testing to guide the selection of opioids.^9^, ^18^, ^19^ Consistent with these recommendations, prior pragmatic trials provided evidence that CYP2D6‐guided opioid therapy in participants with chronic pain could improve pain control.^16^, ^20^, ^21^ However, these guidelines are rarely followed in clinical practice.^22^ The lack of wide adoption provided the rationale for the “A Depression and Opioid Pragmatic Trial in Pharmacogenetics (ADOPT PGx).” The ADOPT PGx Chronic Pain Trial was a large multi‐site prospective, pragmatic, randomized, controlled trial to determine the impact of CYP2D6‐guided opioid prescribing on chronic pain control. This trial aimed to evaluate the utility of CYP2D6 phenotype status, determined by genotyping and drug interactions, in guiding opioid therapy by testing the hypothesis that participants who received CYP2D6‐guided therapy would have better pain control than those who received usual care.

## MATERIALS AND METHODS

### Study design and oversight

ADOPT PGx was designed and conducted by the “Implementing Genomics into Practice Pragmatic Trials Network (IGNITE).”^23^ The ADOPT PGx Chronic Pain trial was part of a Master Protocol Research Program (MPRP) platform trial (NCT04445792) registered on June 22, 2020, before first patient enrollment. In July 2023, each of the three trials under the MPRP was registered under a separate NCT#, and NCT04445792 was converted to a screening record per recent guidance. The ADOPT PGx Chronic Pain trial is registered under NCT05966142, and the trial design has been previously published.^24^ Enrollment began in February 2021, and follow‐up concluded in May 2024. All study aspects were approved by the Duke University Institutional Review Board (IRB) as a single IRB for all clinical sites, with a reliance agreement from each site. All participants provided written, informed consent. An independent data and safety monitoring committee monitored the progress of the trial. The study was regulated under an Abbreviated Investigational Device Exemption due to the genotype‐guided therapy. A patient advisory board assisted in trial design.

### Eligibility criteria, recruitment, and sample collection

#### Inclusion and exclusion criteria

Eligible patients were at least 18 years old, were English‐ or Spanish‐speaking (at some sites), had visited primary care or pain‐related specialty clinics within each enrolling site's healthcare system, reported pain for at least the last 3 months, and were being treated, or considered for treatment, with tramadol, hydrocodone, or codeine for their pain. Hydrocodone, tramadol, and codeine were considered because bioactivation by CYP2D6 can affect their efficacy according to peer‐reviewed clinical guidelines.^9^, ^18^, ^19^ Patients were excluded if they: (1) were unable to provide informed consent, (2) were unable to participate due to cognitive impairment or institutionalization, (3) were too ill to participate as determined by the research staff, (4) had a prior allogeneic stem cell transplant or liver transplant, (5) had a previously known clinical *CYP2D6* pharmacogenetic test, (6) were enrolled in another ADOPT PGx trial, (7) planned to move out of study locales within 6 months of enrollment, (8) were undergoing treatment for active cancer, or (9) had a life expectancy < 12 months.

#### Recruitment

Investigators and research assistants/coordinators discussed the study and recruitment strategies with primary care and specialty clinic leaders and clinicians at each site to identify clinics for participant recruitment. Eligible patients were identified through clinician referral, electronic health record (EHR) review, or direct approach in clinics, and were contacted via phone call or approached in clinics and consented. After providing consent and completing biospecimen collection, participants were randomized to the CYP2D6‐guided or usual care arm.

### Randomization

Participants were randomized 1:1 into the CYP2D6‐guided and usual care arms using computer‐generated random block sizes prepared by an unblinded statistician at the IGNITE Coordinating Center. Randomization was stratified by enrollment site and the presence or absence of depression. Participants randomized to the CYP2D6‐guided arm had an immediate return of *CYP2D6* genotyping results and interpreted “metabolizer” status. Results were returned to the participant's healthcare professional and the EHR (limited only by lab processing time). Participants randomized to the usual care arm had genotyping and return of results delayed until the participant completed the final 6‐month survey.

### Intervention

Pharmacogenetic testing for *CYP2D6* genetic variants was conducted at one of 6 genetic testing laboratories certified by the Clinical Laboratory Improvement Amendments and College of American Pathologists as previously described (**Supplementary Material**).^24^ Participants were classified as PM, IM, NM, or UM using CYP2D6 activity scores derived from the participant's *CYP2D6* genotype and concurrent CYP2D6‐inhibiting medications.^24^ The observed CYP2D6 diplotype frequencies, activity scores, and predicted phenotypes are shown in **Table** **S7**. IM's were defined as those with activity scores > 0 and ≤ 0.75, consistent with previous studies (see Suppl methods page 5).^16^, ^25^ CYP2D6 phenotype–based clinical recommendations have been previously described (**Table** **S1**).^24^ *CYP2D6* genotype results and CYP2D6 phenotypes were transmitted to the EHR through standard laboratory processes at each site. Participants with PM or IM phenotypes were deemed actionable in the trial. For those participants, pharmacogenetic consult notes with CYP2D6‐guided opioid recommendations were included in the EHR at 8 of 10 sites, including information on the CYP2D6 activity score modified by CYP2D6‐interacting drugs. Computerized clinical decision support alerts within the EHR notified prescribers when participants had an actionable *CYP2D6* genotype, including consideration of drug interactions at some clinical sites, when the relevant opioid was ordered. At some sites, participants were able to access their *CYP2D6* genotyping results via their patient portals. There were no restrictions on healthcare professionals or participants discussing their results with each other. Consistent with the pragmatic nature of the study, the participants' healthcare professionals retained full autonomy over all clinical decisions. Participants in the usual care arm had their *CYP2D6* genotype determined and reported in the EHR after all trial participation was completed at 6 months. In both arms, the *CYP2D6* genotyping results were returned to the participants by study personnel after completing the 6‐month survey.

### Data collection and outcomes

Pain control was measured by patient‐reported outcomes using the Patient‐Reported Outcomes Measurement Information System (PROMIS) Scale v1.0–Pain Intensity 3a.^26^ The PROMIS Pain Intensity Scale is a 3‐question survey that measures average pain over the last 7 days, worst pain over the last 7 days, and current pain. Each question uses a 1–5 numeric scale, with a higher score indicating worse pain. The three items were summed to compute the composite pain intensity score (range 3–15). To further evaluate the pain scores between the two arms, we measured average pain intensity using the single‐question PROMIS Numeric Rating Scale v.1.0–Pain Intensity 1a. This survey was administered at baseline and at 1, 3, and 6 months. Baseline surveys were administered by each site's research coordinators, and follow‐up surveys were conducted via telephone or web by the University of Florida College of Pharmacy Call Center, whose staff were masked to the arm of the study. If contact attempts by the call center failed, local research coordinators attempted to reach the participant and administer the follow‐up survey.

The index date for starting follow‐up was when the *CYP2D6* genotyping results were returned to the EHR for the CYP2D6‐guided arm and 1 week after biospecimen collection for the usual care arm. The 1‐week delay for the usual care arm corresponded to the estimated time to return the genotyping results for the CYP2D6‐guided arm. Additional surveys at each time point were administered to assess well‐being, prescription medication misuse, emotional distress, healthcare utilization, productivity loss due to chronic pain, and opioid medication side effects. These results will be reported in future publications.

The pre‐defined primary outcome was pain control, defined as the change in composite pain intensity score from baseline to 3 months using the PROMIS Pain Intensity survey, in the modified intention‐to‐treat (mITT) group. The mITT group was defined as the subset of the intention‐to‐treat (ITT; all randomized participants) group who were successfully genotyped and had an actionable CYP2D6 phenotype. Since the primary endpoint was pain control and not adverse events, UM's were not included in the mITT group. The mITT group comprised the analytical population for the primary and secondary endpoints, unless otherwise specified. Pre‐defined secondary outcomes included pain magnitude and the proportion of participants who achieved clinically significant pain reduction (30% reduction from baseline) at 3 months. Discordant status (i.e., not taking an appropriate opioid for their CYP2D6 phenotype) included CYP2D6 PMs and IMs taking codeine, tramadol, or hydrocodone.

### Sample size estimate

The pilot study indicated a Cohen's D estimate of 0.43 for the effect of pharmacogenetic testing on pain control in a chronic pain population among participants with actionable phenotypes.^16^ A reduced effect size of 0.4 was used for sample size estimation. To achieve 90% power at a significance level of 0.05 with a Cohen's D of 0.40, 134 participants with an actionable phenotype and complete data in each arm were required. We estimated that 10% of participants would have missing data due to discontinuation, loss to follow‐up, or missed visits and that 24% to 33% of participants would have actionable phenotypes, which would require 1233 to 906 randomized participants, respectively.

### Statistical analysis

The ITT population included all participants in the study who were randomized to CYP2D6‐guided or usual care. The difference in the mean change in composite pain intensity score between the CYP2D6‐guided and usual care arms was analyzed using a two‐sample *t*‐test (primary) and covariate‐adjusted linear regression (sensitivity). Continuous secondary outcomes were similarly assessed using a two‐sample *t*‐test and baseline covariate‐adjusted linear regression. Dichotomous secondary outcomes (e.g., drug‐gene concordance) were contrasted using a test of two proportions and baseline covariate‐adjusted logistic regression. Prespecified exploratory time–trend analyses utilized a linear mixed‐effects model to assess the effect of the intervention across all post‐baseline timepoints, with a random effect controlling for repeated measures per participant. Baseline covariates for the single timepoint quantitative endpoints and the time‐trend analyses were age, gender, race, and baseline pain score. Baseline covariates for the dichotomous endpoints were age, race, and baseline pain score. The results are reported as the mean difference (*t*‐test), or least‐squares mean difference (linear regression) with 95% confidence intervals (CIs). The widths of the CIs were not adjusted for multiplicity and should not be used to make a definitive conclusion. Missing data were not imputed because these data were below the prespecified 10% missingness threshold. Post‐hoc analyses of baseline pain measures and change in pain by change in drug‐gene concordance were assessed in the ITT population using a one‐way ANOVA, and pairwise between‐arm comparisons were conducted using Tukey's Honestly Significant Difference; since these analyses were exploratory, they were not corrected for multiple comparisons with the other analyses.

The complete Statistical Analysis Plan is provided in the **Supplementary Material**. All analyses were performed using SAS software, version 9.4 (SAS Institute).

## RESULTS

### Participant enrollment

We attempted to contact 6494 patients and ultimately enrolled 1048 participants (**Figure** **1**). Upon enrollment, 527 participants were randomized to the CYP2D6‐guided arm and 521 to the usual care arm. Overall, 136 participants in the CYP2D6‐guided arm and 146 in the usual care arm had clinically actionable CYP2D6 phenotypes, which met our enrollment target. These two groups (*n* = 282) constituted the mITT group. The demographics of the participants are in **Table** **1**. Approximately half of participants self‐identified as White and a third as Black. The average age was approximately 58 (SD = 11) years, and the majority were female (75%). There were no significant differences in the baseline characteristics between arms, except that a higher proportion of usual care participants had upper‐extremity pain compared with CYP2D6‐guided participants (34.8% vs. 20.6%, respectively).

**Figure 1:**
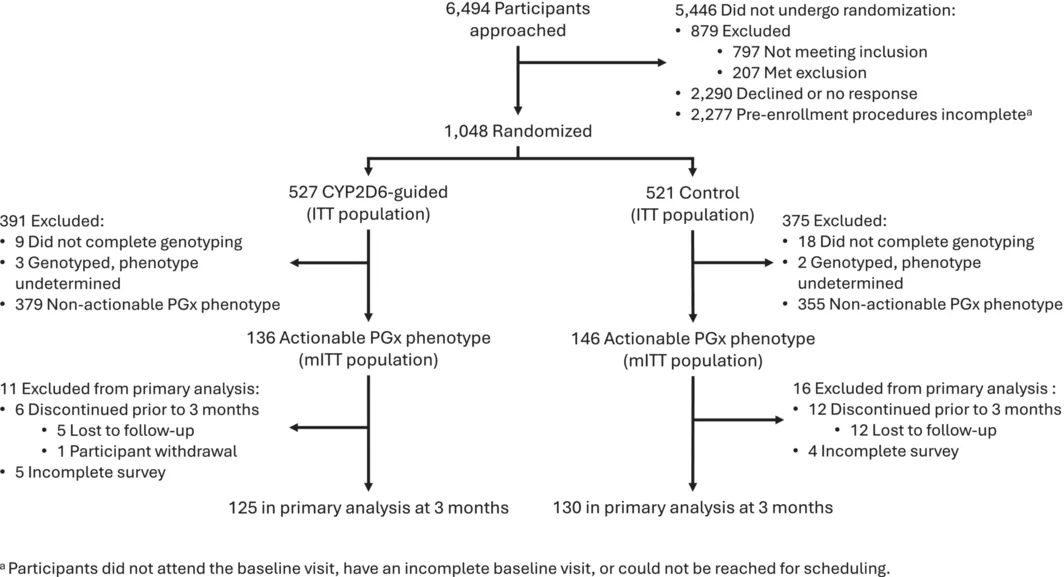
CONSORT diagram.

**Table 1:** Baseline characteristics summary–mITT population

Characteristic	CYP2D6‐guided^a^	Usual care
	(*N* = 136)	(*N* = 146)
Age at randomization (years)		
Mean (SD)	58.0 (10.9)	57.8 (11.3)
Sex at birth, *n* (%)		
Female	95/136 (69.9)	115/146 (78.8)
Male	39/136 (28.7)	31/146 (21.2)
Prefer not to answer	2/136 (1.5)	0/146 (0.0)
Race and ethnicity, *n* (%)		
American Indian, Native American or Alaska Native	2/136 (1.5)	1/146 (0.7)
Asian	2/136 (1.5)	5/146 (3.4)
Black or African American	49/136 (36.0)	48/146 (32.9)
White or European American	66/136 (48.5)	75/146 (51.4)
Hispanic/Latino(a)	11/136 (8.1)	8/146 (5.5)
Other Race	2/136 (1.5)	6/146 (4.1)
Prefer not to answer	1/136 (0.7)	2/146 (1.4)
Unknown or none of these fully describe me	3/136 (2.2)	1/146 (0.7)
Address in medically underserved area^b^, *n* (%)	59/136 (43.4)	62/146 (42.5)
Medical history, *n* (%)		
Diabetes	52/136 (38.2)	54/146 (37.0)
Hypertension	92/135 (68.1)	100/146 (68.5)
Depression	89/136 (65.4)	96/146 (65.8)
Anxiety	75/135 (55.6)	90/146 (61.6)
Psychiatric disorder^c^	15/136 (11.0)	27/146 (18.5)
Cancer	14/136 (10.3)	22/146 (15.1)
BMI		
*n*	134	144
Median (Q1, Q3)	30.8 (25.8, 35.3)	31.4 (27.1, 37.3)
Pain location^d^, *n* (%)		
Lower extremity (legs, upper legs, knees, lower legs, or feet)	65/97 (67.0)	56/92 (60.9)
Lower back	59/97 (60.8)	65/92 (70.7)
Neck	30/97 (30.9)	24/92 (26.1)
Hips	25/97 (25.8)	30/92 (32.6)
Shoulder	24/97 (24.7)	28/92 (30.4)
Upper back	21/97 (21.6)	30/92 (32.6)
Upper extremity (arms, wrist or hands)	20/97 (20.6)	32/92 (34.8)
Head	10/97 (10.3)	10/92 (10.9)
Abdomen	9/97 (9.3)	11/92 (12.0)
Face	6/97 (6.2)	7/92 (7.6)
Chest	3/97 (3.1)	7/92 (7.6)
Other	11/97 (11.3)	12/92 (13.0)
Pain diagnosis^d^, *n* (%)		
Injury or trauma	30/98 (30.6)	25/91 (27.5)
Surgery	11/98 (11.2)	17/91 (18.7)
Rheumatologic disease	28/98 (28.6)	34/91 (37.4)
Musculoskeletal	45/98 (45.9)	47/91 (51.6)
Headaches	5/98 (5.1)	4/91 (4.4)
Nerve damage	11/98 (11.2)	6/91 (6.6)
Other	17/98 (17.3)	17/91 (18.7)

Abbreviations: BMI, body mass index; mITT, modified intention‐to‐treat; SD, standard deviation.

^a^ *P*‐value of 0.029 for upper extremity (comparisons between CYP2D6‐guided and usual care arms), but otherwise non‐significant at < 0.05.

^b^ Excludes participants with addresses that could not be geocoded.

^c^ Psychiatric disorder apart from depression or anxiety.

^d^ Pain location and diagnosis were available for only a subset of participants because collection of these data was initiated after some participants had already been enrolled. Some participants had more than one pain diagnosis and location.

Pain location and diagnoses are shown in **Table** **1**. All regions of the body were reported as sites of pain, with lower back (59.3%) and lower extremities (64.0%) being the most common. The median number of sites of pain recorded per participant was 2.9 of 11 possible locations. The most common pain diagnoses were musculoskeletal pain, injury/trauma, and rheumatologic disease (**Table** **1**). No differences were observed between randomization arms.

### CYP2D6 and opioid use

The CYP2D6 phenotype distribution of the entire study population (ITT; *n* = 1048) is shown in **Table** **2**; these included phenotypes resulting from both *CYP2D6* genotypes and phenoconversion from drugs that inhibit CYP2D6 metabolic activity. Much of the population was NMs (CYP2D6 activity scores > 0.75 to < 2.25; 68.7%), and there were no differences in CYP2D6 phenotypes between arms.

**Table 2:** CYP2D6‐predicted phenotypes, including both genotype and phenoconversion–ITT population

	CYP2D6‐guided	Usual care	*P*
	(*N* = 527)	(*N* = 521)	
Actionable CYP2D6, *n* (%)	136/515 (26.4)	146/501 (29.1)	0.33
CYP2D6 metabolizer status^a^, *n* (%)			0.75
Indeterminant^b^	6/518 (1.2)	4/503 (0.8)	
PM	75/518 (14.5)	70/503 (13.9)	
IM^c^	55/518 (10.6)	73/503 (14.5)	
NM^d^	363/518 (70.1)	338/503 (67.2)	
UM	8/518 (1.5)	7/503 (1.4)	
PM to IM^e^	1/518 (0.2)	1/503 (0.2)	
IM to NM^e^	3/518 (0.6)	2/503 (0.4)	
NM to UM^e^	7/518 (1.4)	8/503 (1.6)	

Abbreviations: IM, intermediate metabolizer; ITT, intention‐to‐treat; NM, normal metabolizer; PM, poor metabolizer; UM, ultrarapid metabolizer.

^a^ 527 randomized to the CYP2D6‐guided arm. Nine individuals were not genotyped, resulting in 518 genotyped individuals.

^b^ Phenotypes could not be determined for these participants. Five participants with indeterminate phenotypes had activity score information, and actionability was determined. Five participants with indeterminate results did not have sufficient activity score information to determine actionability.

^c^ IM was defined as a CYP2D6 activity score of > 0 to ≤ 0.75.

^d^ NM was defined as a CYP2D6 activity score of > 0.75 to < 2.25.

^e^ Participants with a range of possible phenotypes included those with multiple copies of the *CYP2D6* gene and two alleles with different activities, but the genotyping could not determine which allele had multiple copies and thus metabolizer status could not be precisely categorized into one of the phenotypes.

The use of strong and moderate CYP2D6 inhibitors at baseline was common, but not different between arms (**Table** **S2**). The frequencies of genotype‐only‐driven phenotypes are shown in **Table** **S3**. During the trial period, < 1% initiated and 12% stopped a strong CYP2D6 inhibitor, and 1% initiated and 5% stopped a moderate CYP2D6 inhibitor.

The use of opioids at baseline and 3 months in the mITT participants is shown in **Table** **3**. At baseline, tramadol and hydrocodone were the most commonly used opioids; few participants were taking codeine, oxycodone, hydromorphone, methadone, or morphine. Overall, 81.2% of participants were taking one or more of the targeted CYP2D6‐metabolized opioids (tramadol, hydrocodone, or codeine). Less than 1% were taking other opioids. The use of non‐opioids at 3 months is in **Table** **S4**. At 3 months, 73.0% were taking one of the targeted opioids, and approximately 1% were taking the other opioids.

**Table 3:** Pain medication use and drug‐gene concordance – mITT population

	Baseline			3 months		
	CYP2D6‐guided (*N* = 136)	Usual care (*N* = 146)	*P*	CYP2D6‐guided (*N* = 136)	Usual care (*N* = 146)	*P*
Opioid analgesic						
Tramadol	49/136 (36.0)	54/146 (37.0)	0.87	42/123 (34.1)	41/133 (30.8)	0.57
Hydrocodone	60/136 (44.1)	60/146 (41.1)	0.61	47/123 (38.2)	51/133 (38.3)	0.98
Codeine	3/136 (2.2)	6/146 (4.1)	0.50	2/123 (1.6)	5/133 (3.8)	0.45
Oxycodone	0/136 (0.0)	3/146 (2.1)	0.25	2/123 (1.6)	2/133 (1.5)	1.00
Hydromorphone	1/136 (0.7)	0/146 (0.0)	0.48	1/123 (0.8)	0/133 (0.0)	0.48
Methadone	0/136 (0.0)	1/146 (0.7)	1.00	0/123 (0.0)	1/133 (0.8)	1.00
Morphine	0/136 (0.0)	0/146 (0.0)	NA	0/123 (0.0)	1/133 (0.8)	1.00
One or more CYP2D6 opioids, excluding oxycodone^a^	110/136 (80.9)	119/146 (81.5)	0.89	90/123 (73.2)	97/133 (72.9)	0.97
One or more non‐CYP2D6 opioid analgesic	1/136 (0.7)	1/146 (0.7)	1.00	1/123 (0.8)	2/133 (1.5)	1.00
Concordance between opioid analgesic and CYP2D6 phenotype^b^	26/136 (19.1)	27/146 (18.5)	0.89	33/123 (26.8)	36/133 (27.1)	0.97

Abbreviation: mITT, modified intention‐to‐treat.

^a^ Includes tramadol, hydrocodone, or codeine.

^b^ Concordance was defined as the absence of any of the following drug‐gene conflicts between the PGx phenotype and medications reported in medication inventories conducted at baseline and 3 months: an activity score (after accounting for CYP2D6 phenoconversion) ≤ 0.75 and tramadol, hydrocodone, or codeine recorded in the medication inventory.

At baseline, concordance rates in the mITT group were similar between arms at 19.1% and 18.5% for the CYP2D6‐guided and usual care arms, respectively. Likewise, at 3 months, concordance rates were similar at 26.8% and 27.1% in the CYP2D6‐guided and usual care arms, respectively. Thus, the intervention did not impact the drug‐gene concordance.

### Pain scores

The primary endpoint was the composite pain intensity score, where higher values indicate greater pain. The baseline pain intensity scores (mean [SD]) were 10.7 (2.1) and 10.8 (2.4) for the CYP2D6‐guided and usual care arms, respectively. These scores indicate that we enrolled participants with moderate‐to‐high‐impact pain. The pain scores declined slightly from baseline to 3 months for both the CYP2D6‐guided (mean [SD] −0.7 [2.3]) and usual care (mean [SD] −0.8 [2.4]) arms, but this did not differ significantly between arms (0.16, 95% CI −0.42 to 0.74) (**Figure** **2**). Furthermore, the covariate‐adjusted difference in pain scores (least‐squares mean difference −0.03, 95% CI −0.6 to 0.5) and time‐trend and covariate‐adjusted time‐trend analyses were not different between arms (*P* = 0.81, *P* = 0.56, respectively). Additionally, the change in pain magnitude and the proportion of participants who achieved clinically significant pain reduction were not different (**Table** **S5 and** **Table** **S6**).

**Figure 2:**
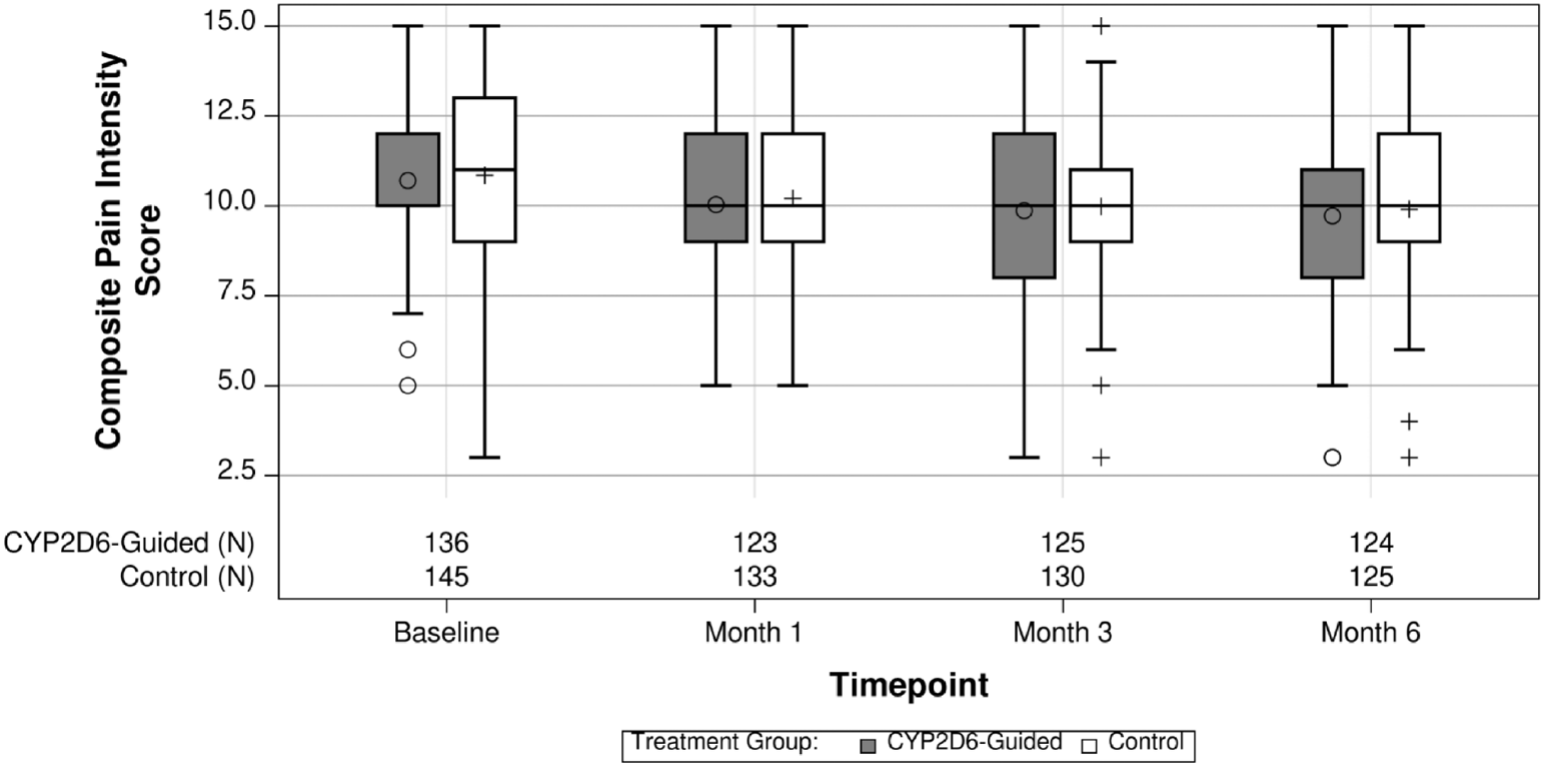
Composite pain intensity score and change in composite pain intensity score, by pharmacogenetic phenotype group, timepoint, and randomized treatment.

Although therapy alignment with the pharmacogenetic recommendations was low in the CYP2D6‐guided arm, some participants' drug‐CYP2D6 phenotype concordance did change between the baseline and 3‐month time points. In a post‐hoc analysis using data from both arms combined, we compared participants who were discordant at baseline and changed to concordant at 3 months (*n* = 28) with those who did not change, including those who remained concordant (*n* = 705) and those who remained discordant (*n* = 201). The change in composite pain scores was non‐significantly greater in the group that changed their medication to align with their CYP2D6 phenotype (discordant to concordant −1.5 [SD = 3.0]) compared with participants whose medication did not change (concordant to concordant −0.6 [SD = 2.3], *P* = 0.14; discordant to discordant −0.6 [SD = 2.2], *P* = 0.13) (**Figure** **S1a**). The change in an alternative pain measure, numeric pain rating, in participants who changed from discordant to concordant (−1.3 [SD = 2.4]) was significantly greater than in the concordant group (−0.3 [SD = 1.9], *P* = 0.01) and discordant group (−0.1 [SD = 1.7], *P* = 0.007) (**Figure** **S1b**). To determine whether adequacy of pain control at baseline may have influenced decisions to change medication, we compared baseline pain scores in these three groups. The baseline composite pain scores were significantly higher in those who changed to concordant (11.8 [SD = 2.6]) compared with the concordant group (10.5 [SD = 2.2], *P* = 0.007) and discordant group (10.5 [SD = 2.1], *P* = 0.02) (**Figure** **S2a**). The numeric pain intensity scores were trending higher in those who changed to concordant (7.7 [SD = 2.4]) compared with those who were discordant (6.8 [SD = 2.2], *P* = 0.098) and those who were concordant (6.8 [SD = 2.1], *P* = 0.077) (**Figure** **S2b**).

## DISCUSSION

This pragmatic intervention trial tested the effect of providing *CYP2D6* genotype and drug interaction guidance on opioid prescription practices for participants with chronic pain. Despite extensive efforts to encourage healthcare professionals to follow the CYP2D6‐guided recommendations, the intervention had a negligible effect on opioid prescribing. Given this lack of effect on opioid prescribing, it is not surprising that no differences were observed in pain control between the CYP2D6‐guided arm and the usual care arm. Changing opioid prescribing in clinical trials has proven similarly challenging in other pragmatic studies that implemented CYP2D6‐guided care. Mosley *et al*. implemented CYP2D6‐guided recommendations for participants with cancer pain, and despite participants reporting high acceptance of pharmacogenetic testing, there was little impact on opioid prescribing.^27^ Smith *et al*. conducted a pragmatic trial (PGx‐ACT) similar to ours in participants with chronic pain, which minimally impacted opioid prescribing;^28^ However, participants whose therapy was changed to align with their CYP2D6 phenotype had improved pain scores. This is consistent with our exploratory analysis findings that showed participants who changed to medications concordant with their CYP2D6 status had improved pain scores. However, these findings need to be interpreted with caution since they were not originally planned and were not corrected for multiple comparisons.

Other studies have shown significant effects on chronic pain control with a CYP2D6‐guided approach. The Smith *et al*. pilot study in chronic pain clinics compared CYP2D6‐guided recommendations with usual care and observed improved composite pain scores in the CYP2D6‐guided clinics (−1.01) versus usual care (−0.40).^16^ Agullo *et al*. showed that participants with chronic pain who received CYP2D6‐guided recommendations, along with *OPRM1* and *COMT* genotyping results, had improved pain intensity (76 vs. 59 mm) and pain relief (28 vs. 48 mm) on visual analogue scales.^21^ Patel *et al*. showed that pharmacogenetic testing resulted in more participants who had at least a 2‐point reduction in their pain score compared with historical controls (56% vs. 30%, respectively).^20^ In a study of participants with cancer pain, those receiving CYP2D6‐guided medications had improved pain control compared with historical controls.^20^ Collectively, these studies support a beneficial effect of providing CYP2D6 opioid guidance if recommendations are followed.

Additionally, retrospective studies support the value of CYP2D6‐guided opioid prescribing to ensure the safety and efficacy of CYP2D6‐metabolized opioids. In the All‐of‐Us cohort, participants who had decreased CYP2D6 activity due to genetics or drug interactions and who were taking hydrocodone, tramadol, or codeine visited the emergency department more often for pain.^29^ Another study that utilized EHR data found that concomitant use of moderate or strong CYP2D6 inhibitors with hydrocodone, oxycodone, tramadol, or codeine was associated with significantly higher rates of emergency department visits for pain.^30^ Both of these studies suggest that impaired CYP2D6 activity reduces the efficacy of these opioids. Similarly, participants with cancer pain who were CYP2D6 IMs or PMs had more hospitalization encounters and were more likely to be treated with later‐line opioids that did not require CYP2D6 activation.^5^ Furthermore, INGENIOUS participants with decreased CYP2D6 activity who were prescribed codeine or tramadol were more likely to report uncontrolled pain.^31^ In addition to pragmatic studies, the Clinical Pharmacogenetics Implementation Consortium (CPIC) reviewed many controlled studies of opioid pharmacokinetics and pharmacodynamics and provided guidelines that support the importance of CYP2D6 metabolizer status in the safety and efficacy of tramadol, hydrocodone, and codeine.^9^

Given the potential benefit of CYP2D6‐guided therapy, it is important to consider the reasons for the lack of success in several trials, including this one, when implementing CYP2D6 guidance for tramadol, hydrocodone, and codeine. Recent policies aimed at reducing opioid exposure may contribute to challenges in conducting chronic pain trials. Data suggest hesitancy to participate in pain trials due to the fear of opioid withdrawal, the lack of access to current therapies, or the return to ineffective therapies.^32^ While we met our enrollment target, we were able to enroll only one of 6 prospective participants approached. Our recruitment strategies involved cold‐calling participants at some sites, and the trial was conducted during the COVID‐19 pandemic; therefore, it is likely that opioid reduction policies may have contributed. These policies included new restrictions on opioid prescribing, and the laws changed in many states where this trial was conducted at the time when study planning was underway. These changes may have impacted trial enrollment and the willingness on the part of prescribers and/or patients to make changes in drug therapy based on CYP2D6 phenotype. Patients with chronic pain often view opioids as critical to their pain management and are reluctant to make changes due to barriers in accessing other pain control strategies, or concern that their pain will worsen if changes are made.^33^ In our study, more than 80% of participants were already on opioids at enrollment. Previous studies have shown that, despite high levels of pain, many patients are satisfied with their pain management,^34^, ^35^ which is consistent with our finding that therapies were not changed, even when the CYP2D6 phenotype data suggested a change was warranted. Thus, implementation of a *CYP2D6* genotyping intervention is likely to be more effective if implemented at the time of opioid therapy initiation. The ongoing C‐PAIN study may provide additional insights into both the recruitment and impact of CYP2D6 status upon study completion.^36^ Additionally, tolerance after long‐term use of opioids may have contributed to the hesitancy of changing therapies. Patients and healthcare professionals recognize that negotiating and maintaining a medication treatment plan in chronic pain is complex and influenced by long‐standing, trusting relationships that overcome barriers, including patients' disempowerment due in part to dismissal of their experiences or inertia in adjustments to treatment plans, which contribute to feelings of futility, thereby impeding any changes.^37^ The reasons for not changing therapies were not ascertained, and unmeasured provider‐, patient‐, or workflow‐related factors may have contributed to the low uptake of recommendations; therefore, we do not know whether the lack of change was the decision of the healthcare professional and/or the participant or a collective decision.

This study has limitations. Because of the pragmatic design, healthcare professionals and participants were allowed to use the recommendations at their discretion, and minimal changes were made that aligned with our recommendations. Most of the eligible participants could not be contacted or declined to participate in the study, and most of the recruitment was done during the COVID‐19 pandemic, which may have generated unknown biases. Genetic variants in other genes that impact opioid response were not included in this trial. We did not assess opioid consumption data, patterns of opioid use, or patient satisfaction with their opioid therapy. Indeed, reductions in opioid dosing without changes in patient‐reported pain would reflect an analgesic effect, which we would not have been able to detect in this trial. Most of the participants were already on opioid therapies, and the benefits of CYP2D6‐guidance may be more impactful when patients initiate opioid therapies. Although recommendations were given to UM's on opioids, since the primary analyses were focused on pain control, we did not include them in the actionable group.

The willingness of healthcare professionals and participants to adjust medications to CYP2D6‐guided recommendations is critical to successfully evaluate the impact of genetic testing in future studies. Therefore, additional scrutiny is needed to identify clinics and professionals ready to adopt clinical decision support recommendations; this may come at the cost of a less pragmatic design or eligibility considerations when enrolling participants, healthcare professionals, or practices. Alternatively, and perhaps preferably, CYP2D6‐guided therapy should be implemented at the time of therapy initiation, not after a period of long‐term treatment with an opioid.

## CONCLUSIONS

Implementation of CYP2D6‐guided opioid therapy did not produce sufficient prescribing changes to assess its impact on pain control in patients with chronic pain. The lack of adherence to the recommendations limits the interpretation of the results relative to the potential clinical benefit of CYP2D6‐guided opioid prescribing for chronic pain. Given the reluctance to change medications, *CYP2D6* genotyping may be most beneficial at the initiation of opioid therapy.

## FUNDING

Research reported in this publication was supported by the National Institutes of Health (NIH) under award numbers U01HG007269, U01HG010232, U01HG010248, U01HG010231, U01HG010245, and U01HG010225, and by the NIH IGNITE Network (https://gmkb.org). KLC is supported by K26DK138374. NIH is the funder, and program officers at the NIH contributed to the study design, monitored its progress, and commented on the manuscript during its preparation. The funders also provided input on the conduct of the study and interpretation of the data but had no role in the collection, management, or analysis of the data. The decision to submit the manuscript for publication was made solely by the authors. Program officers at the National Institutes of Health contributed to the study design, monitored its progress, and reviewed the final manuscript for scientific accuracy.

## CONFLICTS OF INTEREST

The authors declare no competing interests for this work.

## AUTHOR CONTRIBUTIONS

Concept and design: TCS, RAM, RBF, JAJ, HC, ET, MAR, MR, LAO MTE, JFP, SV, CRH, EJC, LHC, LJH, RS, PRD. Acquisition, analysis, or interpretation of data: TCS, RAM, RBF, JTC, MTE, ET, MR, KLC, JAJ, CRH, SS, KAN, MAR, JFP, LAO, HS, RS, AS, PRD. Supervision: TCS, LHC, JFP, CMW, DV, KLC, RS, HC, CRH, LAO, JAJ, PRD. Statistical analysis: RAM, HC. Drafting of the manuscript: TCS, RAM, RBF, JTC, KLC, JAJ, PRD. Critical review of the manuscript for important intellectual content: All authors.

## DISCLAIMER

The content is solely the responsibility of the authors and does not necessarily represent the official views of the National Institutes of Health.

## Supporting information


Data S1.

Data S2.

## Data Availability

De‐identified data and data dictionaries are available via the National Human Genome Research Institute (NHGRI) Genomic Analysis, Visualization and Informatics Lab‐space (AnVIL) (https://anvilproject.org/), ADOPT PGx Chronic Pain Trial [PHS004059v1.p1]. Datasets will be designated as controlled access, and researchers will be able to apply to National Institutes of Health (NIH) data access committees (DACs) for use of these datasets.
