# Supplementary material for: CYP2D6 ‐Guided Opioid Therapy for Chronic Pain: A Multi‐Center Pragmatic Randomized Controlled Trial
Source: Clin Pharmacol Ther. 2026 Oct 15:10.1002/cpt.70505. Online ahead of print. doi: 10.1002/cpt.70505 (PMC13670882; doi:10.1002/cpt.70505)
Supplement: Supplementary file 2 [file CPT-9999-0-s002.docx]

**SUPPLEMENTARY MATERIAL**

**ADOPT PGx Clinical Groups ………………………………………………………………… 2**

**ADOPT PGx Protocol Implementation Teams …......……………………………………….. 2**

**ADOPT PGx Site PIs and Site Study Staff	…………...……………………………………… 3**

**Additional Collaborators ……………………………………………………………………… 4**

**Supplemental Methods ………………………………………………………………………… 5**

**Participating Health Systems ………………………………………………………………….. 5**

**Table S1**. Opioid recommendations based on CYP2D6 phenotype **…………………………….. 6**

**Table S2**. Use of CYP2D6 inhibitors in mITT participants at baseline **………………………… 7**

**Table S3**. CYP2D6-predicted phenotypes based only on genotypes––ITT population **………… 8**

**Table S4**. Use of non-opioid medications for pain control **……………………………………... 9**

**Table S5**. Pain reduction magnitude at 3 months––mITT population **………………………… 10**

**Table S6**. Clinically significant pain reduction (≥30%) at 3 months––mITT population **……... 11**

**Table S7**. *CYP2D6* diplotype counts in the ITT population **…………………………………... 12**

**Figure S1a**. Change in composite pain intensity score by change in drug-gene concordance––ITT population **……...……………………………………………………………. 20**

**Figure S1b.** Change in numeric pain rating by change in drug-gene concordance––ITT population **…...…………………………………………………………………… 20**

**Figure S2a**. Baseline composite pain intensity by change in drug-gene concordance––ITT population **…...…………………………………………………………………… 21**

**Figure S2b.** Baseline numeric pain rating by change in drug-gene concordance––ITT population

**……………………………………………………………………………………. 21**

**References. …………………………………………………………………………………….. 22**

**SUPPLEMENTARY MATERIAL**

**ADOPT PGx Clinical Groups**

Clinical Group Award PI: Duke University Medical Center: Durham, NC: Orlando, L

- Duke University Medical Center, Durham, NC: Voora, D

Clinical Group Award PI: Icahn School of Medicine at Mount Sinai, New York, NY: Horowitz, C

- Icahn School of Medicine at Mount Sinai, New York, NY: Horowitz, C
- The Institute for Family Health, New York, NY: Calman, N

Clinical Group Award PI: Indiana University Health System, Indianapolis, IN: Skaar, T

- Indiana University Health System, Indianapolis, IN: Skaar, T, Dexter, P
- Eskenazi Health, Indianapolis, IN: Skaar, T, Dexter, P

Clinical Group Award PI: University of Florida, Gainesville, FL: Johnson, J, Cavallari, L

- University of Florida, Gainesville, FL: Johnson, J, Cavallari, L
- University of Florida, Jacksonville, FL: Johnson, J, Cavallari, L
- Nemour’s Children’s Health System, Jacksonville, FL: Blake, K
- Nemour’s Children’s Health System, Orlando, FL: Blake, K
- Nemour’s Children’s Health System, Wilmington, DE: Blake, K

Clinical Group Award PI: Vanderbilt University Medical Center, Nashville, TN: Peterson, J

- Vanderbilt University Medical Center, Nashville, TN: Peterson, J, Cavanaugh, K
- Meharry Medical College, Nashville, TN: Singh, R
- Nashville General, Nashville, TN: Singh, R
- Sanford Health, Fargo, ND: Hines, L

IGNITE Coordinating Center: Duke Clinical Research Institute, Durham, NC: Wyatt, C, Chakraborty, H

# **ADOPT PGx Protocol Implementation Teams**

IGNITE Coordinating Center: Hrishikesh Chakraborty (PI), Christina Wyatt (PI), Geoff Ginsburg (*f*-PI), Kady-Ann Steen-Burrell, Bhargav Adagarla, Sarah George, Beth Harris, Jaclyn Holland, Yashika Johnson, Phyllis Kennel, Kristen Linney, Rania Metry, Rachel Myers, Wanda Parker, Gayle Passmore, Carol Pereira, and Teji Rakhra-Burris.

Duke University Medical Center: Deepak Voora (PI), Ryanne Wu, Teji Rakhra-Burris, Azita Sadeghpour (PM), Ruth Lehan, and Tyffany Locklear.

Icahn School of Medicine at Mount Sinai: Carol Horowitz (PI), Michelle Ramos (PM), Sabrina Clermont (PM), Bart Ferket, Kenneth Fifer, Diane Hauser, Joseph Kannry, James Murrough, Aniwaa Owusu Obeng, Janet Seo, Tatiana Sabin, Nandini Shroff, and Saskia Shuman.

Indiana University Health System: Todd C. Skaar (PI), Paul Dexter (PI), Abi Colwell (PM), Amy M. Breman, Zeruesenay Desta, Cathy Fulton, Jennelle C. Hodge, Sheryl Lynch, Jonathan Oliver, Victoria M. Pratt, Ross Robinson, Elizabeth Rowe, Jennifer Stuart, Emma Tillman, and Ashley Vetor.

University of Florida: Julie Johnson (PI), Larisa Cavallari (PI), Alexander Parker (PI), Erica Elwood (PM), Kathryn Blake, Emily Cicali, Kelsey Cook, Karam Diaby, Julio Duarte, Ben Duong, Karla Giron, Elizabeth Eddy, Julia Krutov, Carol Mathews, Haesuk Park, Robyn Nelson, Ryan Rhoden, Caroline Schlierle, Taylor Sullivan, Almut Winterstein, and Kristen Wiisanen.

Vanderbilt University Medical Center: Josh Peterson (PI), Kerri Cavanaugh (PI), Sara Van Driest (*f-*PI), Sara Block (PM), Jordan Baye, Michelle Benck, Chantel Bender, Sylvia Eluhu, Colette Free, Shane Gonnelly, Sarah Hedeen, Lindsay Hines, Steve Houtschilt, Michelle Liu, Salisha Marryshow, Nicole Neville, Natasha Petry, Sidd Pratap, Vernon Sherden, Rajbir Singh, Kimberly Snell, Ana Tomescu, Megan Trietsch, James Walker, Deanna Web, Erin Whiting, and Russell A. Wilke.

National Human Genome Research Institute: Simona Volpi, Sarah Hutchison, Natalie Kucher, Ebony Madden, Jessica Reinach, Renee Rider, Ismail Safi, and Ella Samer.

# **ADOPT PGx Site PIs and Site Study Staff**

Duke University Medical Center: Lori Orlando (PI), Deepak Voora (PI), Azita Sadeghpour, Lani Banez, Lisa Bendz, Jackie Jordan, Ruth Lehan, Tyffany Locklear, Ann McGee, Christina Nix, Lorraine Vergara, and Ryanne Wu.

Icahn School of Medicine at Mount Sinai: Carol Horowitz (PI), Michelle Ramos (PM), Sabrina Clermont (PM), Tyler Colon, Aaishah Francis, Samantha Guagliardo, Najiba Khan, Camila Tan Lam, Michelle Sciarrino, Emma Maiman-Stadtmauer, Sofia Medina-Pardo, Tatiana Sabin, Janet Seo, and Ololade (Lola) Williams.

IGNITE Coordinating Center: Hrishikesh Chakraborty (PI), Christina Wyatt (PI), Geoff Ginsburg (*f*-PI), Sheng Luo, Kady-Ann Steen-Burrell, Kevin Anstrom, Bhargav Adagarla, Alicia Ellis, Stephen Ellis, Sarah George, Beth Harris, Jaclyn Holland, Yashika Johnson, Phyllis Kennel, Hwasoon Kim, Kristen Linney, Peter Merrill, Rania Metry, Rachel Myers, Wanda Parker, Gayle Passmore, Carol Pereira, Teji Rakhra-Burris, Lilin She, Jennifer Shepherd, Saira Siddiqui, and Jun Wen.

Indiana University and Eskenazi Health: Todd C. Skaar (PI), Abi Colwell, Brittany Davis, John Howard, Alia Jamison, Lydia Joyner, Ellie Kiihne, Sheryl Lynch, Johnathan Oliver, Ross Robinson, Libbie Silverman, Jordan Sonneville Jennifer Stuart, Laurel Tokar, and Ashley Vetor.

The Institute for Family Health: Neil Calman (PI), Saskia Shuman, Daviana Buck, Nicole Canchucaja Porshia Cook, Ariel Jacobs Danielle John, Samantha Guagliardo, Diane Hauser, Mirta (Mimi) Milanes, Wambui Ngari, and Nandini Shroff.

Meharry Medical College and Nashville General Hospital: Rajbir Singh (PI), Megan Triestch, Abraham Garcia, Carol Gutierrez, Steven Houtschilt, Maria Lopez, Alan Mejia, Anaite Montes Bu, Deanna Webb, and Sonja White.

Nemours Children’s Health: Kathryn Blake (PI), Ben Duong, Taylor Sullivan, Ali Brewer, Kelsey Cook, Brittni Deadrick, and Julia Krutov.

Sanford Health: Lindsay Hines (PI), Jordan Baye (PI), Colette Free, Michelle Benck, Shane Gonnelly, Sarah Hedeen, Alexis Meyer, and Blair Svennes.

University of Florida at Gainesville: Larisa Cavallari (PI), Erica Elwood (PM), Hana Al Alshaykh, Tala Basha, Emily Cicali, Asia Cobb, Brendon Cooper, Elizabeth Eddy, Amanda Elchynski, Amanda Elsey, Anuksha Gotmare, Karla Giron, Lauren Lemke, Arielle Nelson, Robyn Nelson, Michael Read, Ryan Rhoden, Genevieve Rosier, Isabella Schultz, Chiara Chermont Spina, Joshua Terrell, Alexa Valko, and Precious Williams.

University of Florida at Gainesville Call Center: Tracy Leonard, David Smith, Marian Davis, Vincent Guerra, Jennifer Salo, Claudia Cartaya Torres, Jorge Valedon, and Paola Nieves Viana.

University of Florida at Jacksonville: Alexander Parker (PI), Jose Alonso, Jeffery Gainer, Morgan Howard, Fatoumata Kaba, Brianna Rivers, Liliana Serrano, Kimberly Vigal, and ADeia Williams.

Vanderbilt University Medical Center: Josh Peterson (PI), Kerri Cavanaugh (PI), Sara Van Driest (*f*-PI), Sara Block (PM), Chantel Bender, Angelo Lazaro, Salisha Merryshow, Nicole Neville, Henry Ong, and Erin Whiting.

# **Additional Collaborators**

Amy M. Breman, Anaite Montes Bu, MS, John Thomas Callaghan, Abi Colwell, Raheleh Rahimi Darabad, Daniel Getu, MD, Jennelle C. Hodge, Katherine Huber, MD, Judive Jocelyn, DO, Joseph Kannry, MD, Robin Morman Li, Sheryl Lynch, Rosalena Muckle, MD, Aniwaa Owusu Obeng, Brandi Plunkett, RN, Victoria M. Pratt, Ross Robinson, Siegfried Schmidt, Jennifer Stuart, Evgenia Teal, Petra Townsend, Megan Trietsch, RN, Ashley Vetor, Joshua R. Wellington, and Sonja White, PharmD.

# **Supplemental Methods**

The study start date was February 2, 2021 and completion date was May 10, 2026.

Accepted biospecimen types for genotyping included blood (venipuncture), and for some of the testing laboratories, buccal swabs, mouthwash, or saliva samples were also approved.

Genotyping platforms varied between laboratories but were required to at least include the following alleles: *CYP2D6*2, *3, *4, *5, *6, *8, *9, *10, *17, *29, *41,* and *CYP2D6* copy number. Some laboratories included the following additional alleles *(*7, *13, *31, *34, *39, *45, *46, *58,* and **68*), but the only phenotypes that were changed compared to if they had not included these additional alleles were three participants involving the **7* alleles and one participant involving **31* allele. Intermediate metabolizers (IMs) were defined as having CYP2D6 activity scores of >0 and ≤0.75, consistent with previous studies.^1,2^ This is a more conservative definition than that provided in consensus recommendations from the Clinical Pharmacogenetics Implementation Consortium and Dutch Pharmacogenetics Working Group, which define the IM phenotype as having an activity score of >0 and <1.25.^3^ There is some uncertainty about the CYP2D6 activity score of IMs that should be the cutoff for making altered opioid recommendations; we used a more conservative definition of IM given results in support of a CYP2D6-guided approach to pain management from the studies referenced above, which defined IMs using the lower score range. There were 142 participants with an activity score of 1.0 in the CYP2D6-guided group and 123 participants in the control group; since we did not provide recommendations for these participants, we are not able to determine if expanding the IM group to include those with an activity score of 1.0 would have had any impact.

As defined by the United States Food and Drug Administration, moderate CYP2D6 inhibitors included duloxetine, mirabegron, terbinafine, abiraterone, cinacalcet, and lorcaserin. Strong inhibitors included paroxetine, bupropion, fluoxetine, and quinidine. No participants were on cinacalcet, lorcaserin, or quinidine.

# **Participating Health Systems**

The study was conducted at the following sites: Duke University Health System, Eskenazi Health, Indiana University Health, Institute for Family Health at Mount Sinai Health System, Meharry Medical College, Mount Sinai Health System, Sanford Health, University of Florida Health in Gainesville and Jacksonville, and Vanderbilt University Medical Center with a network Coordinating Center at Duke University.

# **Table S1**. Opioid recommendations based on CYP2D6 phenotype

# **Table S2**. Use of CYP2D6 inhibitors in mITT participants at baseline

|  | **CYP2D6-guided**  **(N=136)** | **Usual care (N=146)** |
| --- | --- | --- |
| Strong CYP2D6 inhibitors, n (%) |  |  |
| Fluoxetine | 23 (16.9) | 15 (10.3) |
| Paroxetine | 5 (3.7) | 4 (2.7) |
| Bupropion | 33 (24.3) | 37 (25.3) |
| Terbinafine | 1 (0.7) | 1 (0.7) |
| Moderate CYP2D6 inhibitors, n (%) |  |  |
| Duloxetine | 38 (27.9) | 47 (32.2) |
| Mirabegron | 6 (4.4) | 1 (0.7) |
| mITT, modified intention-to-treat. | | |

# **Table S3**. CYP2D6-predicted phenotypes based only on genotypes––ITT population

| **CYP2D6 metabolizer status** | **CYP2D6-guided**   **(N=527)** | **Usual care**  **(N=521)** |
| --- | --- | --- |
| Indeterminant | 5/518 (1.0) | 2/503 (0.4) |
| PM | 20/518 (3.9) | 21/503 (4.2) |
| IM | 40/518 (7.7) | 51/503 (10.1) |
| NM | 430/518 (83.0) | 408/503 (81.1) |
| UM | 10/518 (1.9) | 9/503 (1.8) |
| PM to IM | 1/518 (0.2) | 1/503 (0.2) |
| IM to NM | 4/518 (0.8) | 1/503 (0.2) |
| IM to UM | 0/518 (0.0) | 0/503 (0.0) |
| NM to UM | 8/518 (1.5) | 10/503 (2.0) |

IM, intermediate metabolizer; NM, normal metabolizer; PM, poor metabolizer; ITT, intention-to-treat; UM, ultrarapid metabolizer.

**Table S4**. Use of non-opioid medications for pain control

| **Medication** | **CYP2D6-guided**  **(N=136)** | **Usual care (N=146)** | ***P*-value** |
| --- | --- | --- | --- |
| Tylenol | 67 (49.3) | 79 (54.1) | 0.42 |
| Aspirin | 31 (22.8) | 25 (17.1) | 0.23 |
| NSAID | 52 (38.2) | 62 (42.5) | 0.47 |
| Gabapentinoids | 66 (48.5) | 63 (43.2) | 0.37 |
| SSRI class medication | 43 (31.6) | 33 (22.6) | 0.09 |
| SNRI class medication | 42 (30.9) | 52 (35.6) | 0.40 |
| TCA class medication | 11 (8.1) | 8 (5.5) | 0.38 |

NSAID, non-steroidal anti-inflammatory drug; SNRI, serotonin-norepinephrine reuptake inhibitor; SSRI, selective serotonin reuptake inhibitor; TCA, tricyclic antidepressant.

# **Table S5**. Pain reduction magnitude at 3 months––mITT population

|  | **CYP2D6-guided** | **Usual care** | ***P*-value^a^** | **Covariate-adjusted P-value^b^** |
| --- | --- | --- | --- | --- |
| Pain reduction magnitude^c^ | 0.94 (0.32) | 0.97 (0.57) | 0.68 | 0.22 |

mITT, modified intention-to-treat.

^a^Treatment arm difference assessed using a two-sample *t*-test of the log-transformed ratio of pain at 3 months relative to baseline.

^b^Treatment arm difference assessed using multivariable linear regression of the log-transformed ratio of pain at 3 months relative to baseline. Model adjusted for age, gender, race, and baseline pain score.

^c^Values are the means and standard deviations of the reduction in composite pain scores from baseline to 3 months.

# **Table S6**. Clinically significant pain reduction (≥30%) at 3 months––mITT population

|  | **CYP2D6-guided** | **Usual care** | ***P*-value** | **Covariate-adjusted *P*-value** |
| --- | --- | --- | --- | --- |
| Clinically significant pain reduction^a^, % | 13.6 | 20.9 | 0.12 | 0.17 |

mITT, modified intention-to-treat.

^a^Values are the percentage of participants with ≥30% reduction in composite pain intensity scores.

**Table S7**. *CYP2D6* diplotype counts in the ITT population

| **Genotype** | **Count** | **Activity Score** |  | **Protocol Phenotype** |  |
| --- | --- | --- | --- | --- | --- |
| (*1/*1)xN | 1 | 3.00 |  | ultrarapid metabolizer |  |
| (*1/*10)2N | 1 | 1.25 |  | normal metabolizer |  |
| (*1/*17)xN | 1 | 2.00 |  | normal metabolizer |  |
| (*1/*2)xN | 2 | 3.00 |  | ultrarapid metabolizer |  |
| (*1/*29)xN | 1 | 2.00-2.50 |  | normal to ultrarapid metabolizer |  |
| (*1/*2A)x3 | 1 | 3.00 |  | ultrarapid metabolizer |  |
| (*1/*4)5N | 1 | 1.00-4.00 |  | normal to ultrarapid metabolizer |  |
| (*1/*4)x3 | 1 | 1.00 |  | normal metabolizer |  |
| (*1/*4)x4 | 1 | 1.00-3.00 |  | normal to ultrarapid metabolizer |  |
| (*1/*4)xN | 2 | 1.00-≥2.00 |  | normal metabolizer; normal to ultrarapid metabolizer |  |
| (*1/*7)xN^a,b^ | 1 | 1.00-≥2.00 |  | normal to ultrarapid metabolizer |  |
| (*10/*10)2N | 2 | 0.50; 1.00 |  | intermediate metabolizer; normal metabolizer |  |
| (*10/*17)2N | 2 | 0.75 |  | intermediate metabolizer |  |
| (*10/*41)2N | 2 | 0.75 |  | intermediate metabolizer |  |
| (*2/*10)2N | 2 | 1.25 |  | normal metabolizer |  |
| (*2/*10)xN | 1 | 1.50-2.25 |  | normal to ultrarapid metabolizer |  |
| (*2/*17)xN | 1 | 2.00-≥2.00 |  | normal to ultrarapid metabolizer |  |
| (*2/*2)xN | 2 | 3.00 |  | ultrarapid metabolizer |  |
| (*2/*2A)x3 | 2 | 3.00 |  | ultrarapid metabolizer |  |
| (*2/*4)x3 | 1 | 1.00-2.00 |  | normal metabolizer |  |
| (*2/*4)xN | 1 | 1.00-2.00 |  | normal metabolizer |  |
| continued | | | | | |

| **Genotype** | | **Count** | **Activity Score** |  | **Protocol Phenotype** | | |
| --- | --- | --- | --- | --- | --- | --- | --- |
| (*2A/*17)x3 | | 1 | 2.00-2.50 |  | normal to ultrarapid metabolizer | | |
| (*2A/*2A)x3 | | 1 | 3.00 |  | ultrarapid metabolizer | | |
| (*2A/*3)x3 | | 1 | 1.00-2.00 |  | normal metabolizer | | |
| (*2A/*4)x3 | | 1 | 1.00-2.00 |  | normal metabolizer | | |
| (*4/*10)2N | | 2 | 0.25 |  | intermediate metabolizer | | |
| (*4/*10)x3 | | 1 | 0.25-0.50 |  | intermediate metabolizer | | |
| (*4/*10)xN | | 1 | 0.25 |  | intermediate metabolizer | | |
| (*4/*17)xN | | 2 | 0.50-1.00 |  | intermediate to normal metabolizer | | |
| (*4/*29)xN | | 1 | 0.50 |  | intermediate to normal metabolizer | | |
| (*4/*4)x3 | | 2 | 0.00 |  | poor metabolizer | | |
| (*4/*6)xN | | 1 | 0.00 |  | poor metabolizer | | |
| (*41/*41) 2N | | 1 | 1.00 |  | normal metabolizer | | |
| (2A*/*17)2N | | 1 | 1.50 |  | normal metabolizer | | |
| (2A/5)1N | | 1 | 1.00 |  | normal metabolizer | | |
| *1/*1 | | 126 | 2.00 |  | normal metabolizer | | |
| *1/*1/xN | | 1 | 3.00 |  | ultrarapid metabolizer | | |
| *1/*10 | | 10 | 1.25 |  | normal metabolizer | | |
| *1/*13^a^ | | 1 | 1.00 |  | normal metabolizer | | |
| *1/*17 | | 49 | 1.50 |  | normal metabolizer | | |
| *1/*17,DUP | | 1 | 2.00 |  | normal metabolizer | | |
| *1/*1x2 | | 1 | 3.00 |  | ultrarapid metabolizer | | |
| *1/*2 | | 119 | 2.00 |  | normal metabolizer | | |
| continued | | | | | | |  |
|  | | | | | | |  |

| **Genotype** | **Count** | **Activity Score** |  | **Protocol Phenotype** | |
| --- | --- | --- | --- | --- | --- |
| *1/*2 | 1 | 2.00 |  | normal metabolizer | |
| *1/*2,DUP | 2 | 3.00 |  | ultrarapid metabolizer | |
| *1/*29 | 28 | 1.50 |  | normal metabolizer | |
| *1/*29 DUP | 1 | 2.00-≥2.00 |  | normal to ultrarapid metabolizer | |
| *1/*2A | 31 | 2.00 |  | normal metabolizer | |
| *1/*3 | 8 | 1.00 |  | normal metabolizer | |
| *1/*34^a^ | 1 | 2.00 |  | normal metabolizer | |
| *1/*39^a^ | 1 | 2.00 |  | normal metabolizer | |
| *1/*4 | 77 | 1.00 |  | normal metabolizer | |
| *1/*41 | 43 | 1.25; 1.50 |  | normal metabolizer | |
| *1/*4Nx2n-*4 | 1 | 1.00 |  | normal metabolizer | |
| *1/*5 | 29 | 1.00 |  | normal metabolizer | |
| *1/*58^a^ | 1 | 1.00 |  | indeterminate | |
| *1/*6 | 3 | 1.00 |  | normal metabolizer | |
| *1/*68-*4^a^ | 2 | 1.00 |  | normal metabolizer | |
| *1/*7^a^ | 1 | 1.00 |  | normal metabolizer | |
| *1/*9 | 22 | 1.25; 1.50 |  | normal metabolizer | |
| *10/*10 | 1 | 0.50 |  | intermediate metabolizer | |
| *10/*17 | 4 | 0.75 |  | intermediate metabolizer | |
| *10/*17,DUP | 1 | 1.00 |  | normal to ultrarapid metabolizer | |
| *10/*29 | 4 | 0.75 |  | intermediate metabolizer | |
| *10/*29(1N each) | 1 | >0.00-0.75 |  | poor to intermediate metabolizer | |
| continued | | | | |  |
|  | | | | |  |

| **Genotype** | **Count** | **Activity Score** |  | **Protocol Phenotype** | |
| --- | --- | --- | --- | --- | --- |
| *10/*40 | 1 | 0.25 |  | intermediate metabolizer | |
| *10/*41 | 1 | 0.50 |  | intermediate metabolizer | |
| *10/*58 | 1 | 0.25 |  | indeterminate | |
| *13-*2/*41^a^ | 1 | 1.50 |  | normal metabolizer | |
| *17/*17 | 12 | 1.00 |  | normal metabolizer | |
| *17/*29 | 18 | 1.00 |  | normal metabolizer | |
| *17/*29 X3N | 1 | 1.50 |  | normal metabolizer | |
| *17/*41 | 2 | 1.00 |  | normal metabolizer | |
| *1x2/*2A or *1/*2Ax2 | 1 | 3.00 |  | ultrarapid metabolizer | |
| *1xN/*2 | 1 | 3.00 |  | ultrarapid metabolizer | |
| *2/*10 | 13 | 1.25; 1.50 |  | normal metabolizer | |
| *2/*13^a^ | 1 | 1.00 |  | normal metabolizer | |
| *2/*17 | 15 | 1.50 |  | normal metabolizer | |
| *2/*17 X3n | 1 | 2.00-2.50 |  | normal to ultrarapid metabolizer | |
| *2/*2 | 38 | 2.00 |  | normal metabolizer | |
| *2/*2 Xn | 2 | 3.00-4.00 |  | ultrarapid metabolizer | |
| *2/*2,DUP | 1 | 3.00 |  | ultrarapid metabolizer | |
| *2/*29 | 8 | 1.50 |  | normal metabolizer | |
| *2/*29 X3n | 2 | 2.00-2.50 |  | normal to ultrarapid metabolizer | |
| *2/*29 Xn | 1 | 2.00-3.50 |  | normal to ultrarapid metabolizer | |
| *2/*29x2 | 1 | 1.50 |  | normal metabolizer | |
| *2/*3 | 2 | 1.00 |  | normal metabolizer | |
| continued | | | | |  |
|  | | | | |  |

| **Genotype** | **Count** | **Activity Score** |  | **Protocol Phenotype** | |
| --- | --- | --- | --- | --- | --- |
| *2/*4 | 46 | 1.00 |  | normal metabolizer | |
| *2/*4 X3n | 1 | 1.00 |  | normal metabolizer | |
| *2/*4 Xn | 3 | 1.00-3.0 |  | normal to ultrarapid metabolizer | |
| *2/*4/xN | 2 | 2.00 |  | normal metabolizer | |
| *2/*41 | 26 | 1.25; 1.50 |  | normal metabolizer | |
| *2/*41 DUP | 1 | 2.00 |  | normal to ultrarapid metabolizer | |
| *2/*5 | 16 | 1.00 |  | normal metabolizer | |
| *2/*58 | 1 | 1.00 |  | normal metabolizer | |
| *2/*6 | 1 | 1.00 |  | normal metabolizer | |
| *2/*68+*4^a^ | 1 | 1.00 |  | normal metabolizer | |
| *2/*9 | 6 | 1.50 |  | normal metabolizer | |
| *29/*29 | 3 | 1.00 |  | normal metabolizer | |
| *29/*41 | 2 | 1.00 |  | normal metabolizer | |
| *29X2n/*40 | 1 | 1.00 |  | normal metabolizer | |
| *2A/*10 | 1 | 1.25 |  | normal metabolizer | |
| *2A/*10(1N for Exon 9) | 1 | 0.25 |  | intermediate metabolizer | |
| *2A/*17 | 3 | 1.50 |  | normal metabolizer | |
| *2A/*2 | 1 | 2.00 |  | normal metabolizer | |
| *2A/*29 | 1 | 1.50 |  | normal metabolizer | |
| *2A/*2A | 9 | 2.00 |  | normal metabolizer | |
| *2A/*3 | 2 | 1.00 |  | normal metabolizer | |
| *2A/*4 | 7 | 1.00 |  | normal metabolizer | |
| continued | | | | |  |
|  | | | | |  |

| **Genotype** | **Count** | **Activity Score** |  | **Protocol Phenotype** | |
| --- | --- | --- | --- | --- | --- |
| *2A/*41 | 5 | 1.50 |  | normal metabolizer | |
| *2A/*5 | 1 | 1.00 |  | normal metabolizer | |
| *2A/*9 | 2 | 1.50 |  | normal metabolizer | |
| *2AxN/*17 | 1 | 2.50 |  | ultrarapid metabolizer | |
| *3/*4 | 3 | 0.00 |  | poor metabolizer | |
| *3/*41 | 1 | 0.50 |  | intermediate metabolizer | |
| *3/*5 | 1 | 0.00 |  | poor metabolizer | |
| *3/*9 | 1 | 0.25 |  | intermediate metabolizer | |
| *31/*31^a,b^ | 1 | 0.00 |  | poor metabolizer | |
| *4/*10,DUP | 1 | 0.25 |  | intermediate metabolizer | |
| *4/*17 | 12 | 0.50 |  | intermediate metabolizer | |
| *4/*17 X3n | 1 | 0.50-1.00 |  | intermediate to normal metabolizer | |
| *4/*29 | 4 | 0.50 |  | intermediate metabolizer | |
| *4/*29 X3n | 1 | 0.50-1.00 |  | intermediate to normal metabolizer | |
| *4/*4 | 17 | 0.00 |  | poor metabolizer | |
| *4/*4 X4n | 1 | 0.00 |  | poor metabolizer | |
| *4/*4/xN | 1 | 0.00 |  | poor metabolizer | |
| *4/*41 | 23 | 0.50 |  | intermediate metabolizer | |
| *4/*4M | 1 | 0.00 |  | poor metabolizer | |
| *4/*4xN | 1 | 0.00 |  | poor metabolizer | |
| *4/*5 | 7 | 0.00 |  | poor metabolizer | |
| *4/*6 | 1 | 0.00 |  | poor metabolizer | |
| continued | | | | |  |
|  | | | | |  |

| **Genotype** | **Count** | **Activity Score** |  | **Protocol Phenotype** | |
| --- | --- | --- | --- | --- | --- |
| *4/*68-*4^a^ | 2 | 0.00 |  | poor metabolizer | |
| *4/*9 | 4 | 0.50 |  | intermediate metabolizer | |
| *41/*41 | 11 | 1.00 |  | normal metabolizer | |
| *41/*68-*4^a^ | 1 | 0.25 |  | intermediate metabolizer | |
| *4x2N/*34^a^ | 1 | 1.00 |  | normal metabolizer | |
| *5/*10 | 4 | 0.25; 0.50 |  | intermediate metabolizer | |
| *5/*17 | 7 | 0.50 |  | intermediate metabolizer | |
| *5/*29 | 2 | 0.50 |  | intermediate metabolizer | |
| *5/*41 | 2 | 0.50 |  | intermediate metabolizer | |
| *5/*45^a^ or *5/*46^a^ | 1 | 1.00 |  | normal metabolizer | |
| *5/*5 | 1 | 0.00 |  | poor metabolizer | |
| *5/*6 | 1 | 0.00 |  | poor metabolizer | |
| *5/*9 | 1 | 0.25 |  | intermediate metabolizer | |
| *6/*10 | 1 | 0.25 |  | intermediate metabolizer | |
| *6/*17 | 1 | 0.50 |  | intermediate metabolizer | |
| *6/*41 | 2 | 0.25; 0.50 |  | intermediate metabolizer | |
| *6/*9 | 1 | 0.50 |  | intermediate metabolizer | |
| *7/*41^a,b^ | 1 | 0.50 |  | intermediate metabolizer | |
| *9/*10 | 1 | 0.75 |  | intermediate metabolizer | |
| *9/*29 | 2 | 1.00 |  | normal metabolizer | |
| *9/*41 | 2 | 1.00 |  | normal metabolizer | |
| *9/*9 | 1 | 1.00 |  | normal metabolizer | |
| continued | | | | |  |
|  | | | | |  |

| **Genotype** | **Count** | **Activity Score** |  | **Protocol Phenotype** | |
| --- | --- | --- | --- | --- | --- |
| 1/*4 X3n | 1 | 1.00-2.00 |  | normal metabolizer | |
| 1/novel allele with c.1322G>A, p.Arg441His | 1 | 1.00-2.00 |  | normal metabolizer | |
|  |  |  |  |  | |
| Indeterminate | 4 |  |  | indeterminate | |
| Unable to Genotype | 1 |  |  | indeterminate | |
|  | | | | |  |
| Note: Cases where there are multiple options for the activity scores resulted from either changes in the activity score of the allele during the trial (e.g. *10) or variation in the activity scores used between labs. There was only one participant whose *CYP2D6* genotype-determined phenotype assignment was different due to the change; it was the participant with the *(*10/*10)2N* genotype who was assigned a CYP2D6 activity score of 1.00 (normal metabolizer). The change in activity score from 0.5 to 0.25 for the **10* allele resulted in a change in the total activity score for this participant from 1.00 to 0.5.  Any genotypes without the copy numbers (e.g. *xN, 2N*, etc) or a **5* genotype were assumed to have a copy number of 2. The labs used by different sites used different allele nomenclature for the copy numbers, resulting in differing names assigned for the same genotypes. For example, **2/*2 Xn* is the same as **2/*2,DUP*). To show the differences observed in this pragmatic trial, the actual names used by the labs are retained in this table, even though some represent the same genotypes. Prior to the trial, some of the differing allele names from different laboratories representing the allele were haromonized into one allele name, but other new designations were reported during the trial and were included here as reported by the laboratory.  The activity scores that are listed are those that were either reported by the laboratories and/or determined at the time of the trial and thus used in the trial.  ^a^Denotes *alleles that were included in the assays by some but not all laboratories and was not required to be included by the protocol.  ^b^Denotes diplotypes that had phenotypes impacted by the **7* allele that was included in the assays by some but not all laboratories. | | | | |  |

# **a.**

# **b.**

**Figure S1. a.** Change in composite pain intensity score by change in drug-gene concordance––ITT population. **b.** Change in numeric pain rating by change in drug-gene concordance––ITT population. ITT, intention-to-treat.

# **a.**

# **b.**

**Figure S2. a.** Baseline composite pain intensity by change in drug-gene concordance––ITT population **b.** Baseline numeric pain rating by change in drug-gene concordance––ITT population. ITT, intention-to-treat.

**References**:

^1^Thomas CD, Parvataneni HK, Gray CF, Deen JT, Prieto HA, Pulido LF, Elsey AR, Elwood EN, Starostik P, Gong Y, Fillingim RB, Johnson JA, Cavallari LH. A hybrid implementation-effectiveness randomized trial of CYP2D6-guided postoperative pain management. Genet Med. 2021 Apr;23(4):621-628. doi: 10.1038/s41436-020-01050-4. Epub 2021 Jan 8. PMID: 33420349; PMCID: PMC8525217.

^2^Smith DM, Weitzel KW, Elsey AR, Langaee T, Gong Y, Wake DT, Duong BQ, Hagen M, Harle CA, Mercado E, Nagoshi Y, Newsom K, Wright A, Rosenberg EI, Starostik P, Clare-Salzler MJ, Schmidt SO, Fillingim RB, Johnson JA, Cavallari LH. CYP2D6-guided opioid therapy improves pain control in CYP2D6 intermediate and poor metabolizers: a pragmatic clinical trial. Genet Med. 2019 Aug;21(8):1842-1850. doi: 10.1038/s41436-018-0431-8. Epub 2019 Jan 23. PMID: 30670877; PMCID: PMC6650382.

^3^Caudle KE, Sangkuhl K, Whirl-Carrillo M, Swen JJ, Haidar CE, Klein TE, Gammal RS, Relling MV, Scott SA, Hertz DL, Guchelaar HJ, Gaedigk A. Standardizing CYP2D6 Genotype to Phenotype Translation: Consensus Recommendations from the Clinical Pharmacogenetics Implementation Consortium and Dutch Pharmacogenetics Working Group. Clin Transl Sci. 2020 Jan;13(1):116-124. doi: 10.1111/cts.12692. Epub 2019 Oct 24. PMID: 31647186; PMCID: PMC6951851.
